# Risk factors for portal vein thrombosis or venous thromboembolism in a large cohort of hospitalized cirrhotic patients

**DOI:** 10.1007/s11739-022-02928-8

**Published:** 2022-01-25

**Authors:** Mariella Faccia, Francesco Santopaolo, Antonio Gasbarrini, Maurizio Pompili, Maria Assunta Zocco, Francesca Romana Ponziani

**Affiliations:** 1 Internal Medicine, SS Annunziata Hospital, Sulmona ASL1, Abruzzo, Italy; 2 Internal Medicine and Gastroenterology, Hepatology Unit, Fondazione Policlinico Universitario Agostino Gemelli IRCCS, Rome, Italy; 3 Catholic University of the Sacred Heart, Rome, Italy

**Keywords:** Hepatic encephalopathy, Portal hypertension, HCC, Anticoagulant, Cancer, Diabetes

## Abstract

**Background:**

Portal vein thrombosis (PVT) and venous thromboembolism (VTE) are fearsome complications of liver cirrhosis.

**Objectives:**

To assess the prevalence and the main risk factors for venous thrombotic complications in hospitalized cirrhotic patients.

**Patients/methods:**

We retrospectively reviewed electronic administrative discharge data of 19461 cirrhotic patients hospitalized over a 35-year period; univariate and multivariate logistic regression was used to asses risk factors for PVT or VTE and their impact on hospital stay and mortality.

**Results:**

382 out of 7445 patients (5.1%) were diagnosed with PVT and 95 (1.3%) with VTE. Liver cirrhosis complications were observed in 45% of patients. Hepatic encephalopathy (HE) (OR 13.88 [10.76–17.98] *p* < 0.0001), endoscopic signs of portal hypertension (OR 1.33 [1.02–1.75] *p* = 0.02), hepatocellular carcinoma (HCC) (OR 4.59 [3.6–5.84] *p* < 0.0001), diabetes (OR 1.68 [1.27–2.22] *p* = 0.0001), abdominal surgery/invasive procedures (OR 2.03 [1.56–2.64] *p* < 0.0001) emerged as independent predictors of PVT. Higher risk of VTE was observed in patients with HE (OR 3.21 [1.78–5.79] *p* < 0.0001), HCC (OR 1.98 [1.23–3.19] *p* = 0.002) or other tumors (OR 2.48 [1.42–4.32] *p* = 0.001), acute illnesses (infections OR 3.01 [1.84–5.05] *p* = 0.0001; cardiac/respiratory insufficiency OR 2.4 [1.27–4.53] *p* = 0.003; acute myocardial infarction/stroke OR 7.86 [1.76–35.12] *p* = 0.003). VTE was the only independent predictor of in-hospital mortality (OR 4.45 [1.05–18.81] *p* = 0.042).

**Conclusions:**

Liver disease complications related to portal hypertension, HCC or other tumors, diabetes, acute illnesses (i.e. infections, cardiac/pulmonary insufficiency, acute myocardial infarction/stroke) and abdominal interventions are associated with increased risk of PVT or VTE in hospitalized cirrhotic patients, and should be considered to define personalized preemptive approaches.

**Supplementary Information:**

The online version contains supplementary material available at 10.1007/s11739-022-02928-8.

## Introduction

Portal vein thrombosis (PVT) and venous thromboembolism (VTE) are fearsome complications of liver cirrhosis, resulting from complex changes in haemodynamic and haemostatic pathways caused by liver dysfunction [1, 2]. PVT prevalence ranges from 1% in patients with compensated liver disease, to 40% in those affected by hepatocellular carcinoma (HCC) and to 8–25% in liver transplant (LT) candidates, while VTE has been reported in up to 0.8–7% of hospitalized cirrhotic patients [3–8]. Thrombotic complications have a negative impact on prognosis; in particular, increased mortality rates have been reported in patients with liver cirrhosis and deep vein thrombosis (DVT) or pulmonary embolism (PE) [9]. A worse outcome has also been associated with VTE in patients with decompensated cirrhosis compared to compensated disease [10]. However, the prognostic value of PVT on liver disease progression and outcome remains an unresolved question, because clinical trials are based on small cohorts of patients with short follow-up, and randomized controlled trials are lacking [11]. Progression or regression of partial PVT also does not seem to have a clear impact on the natural history of liver cirrhosis. It has been reported that improvement of PVT provided no benefit on cirrhosis-related complications and survival, whereas only Child–Pugh score was found to be an independent predictor of mortality and liver failure in this patient population [11]. In contrast, PVT, when complete and extended to the distal superior mesenteric vein, was associated with higher short-term and medium-term mortality after LT [12].

The occurrence of thrombotic complications in patients with liver cirrhosis is difficult to predict. Conventional coagulation tests are of limited value when estimating the haemostatic balance, and even misleading when assessing life-threatening bleeding risk [1]. Given the negative prognostic impact of thrombotic complications in liver cirrhosis, the identification of reliable predictive factors is crucial for clinical decision-making, and for the management of thromboprophylaxis [9]. In addition, comorbidity scores have been validated as predictors of mortality among patients with liver cirrhosis and LT recipients [13, 14]. Although comorbidities have shown a significant influence on coagulation balance and are commonly included in thrombosis prediction scoring systems, their role in the specific haemostatic setting of liver cirrhosis has not been explored yet.

The main aim of this study was to define the prevalence of PVT and VTE in a large population of hospitalized cirrhotic patients, and to investigate whether factors related to liver disease and comorbidities could increase the risk of thrombotic events.

## Patients and methods

We retrospectively reviewed electronical records of all patients discharged from the Fondazione Policlinico Universitario Agostino Gemelli IRCCS in Rome over a 35-year period (1982–2017) with a diagnosis of: “liver cirrhosis”, “alcoholic liver cirrhosis” or “cirrhosis of liver without mention of alcohol”. The review was conducted by two independent researchers (MF and FRP) and based on international classification of diseases 9th revision (ICD-9) codes (Supplementary Table 1). To avoid coding errors, we included only patients with at least another hospital admission with the same ICD-9 code in the following 18-month period, or those with one or more further ICD-9 codes suggestive of liver cirrhosis complications, such as “hepatic encephalopathy” (HE), “endoscopic signs of portal hypertension” (including “esophageal varices without bleeding”, “esophageal varices with bleeding”), “ascites”, and “hepatorenal syndrome” (HRS). To avoid the inclusion of patients without liver cirrhosis, the ICD-9 codes “biliary cirrhosis” and “chronic liver disease” were not considered. We also excluded patients with age < 18 years.

In this population, we searched for the occurrence of non-neoplastic PVT or VTE, the latter including “deep vein thrombosis”, “pulmonary embolism” and thrombosis of the “inferior vena cava” (IVC). Phlebitis and thrombophlebitis of superficial vessels and catheter-related venous thrombosis were excluded. In case of multiple admissions per patient, we included only the first admission or the first hospitalization with a discharge diagnosis of thrombotic events.

Three subgroups were identified based on the prevalence of thrombotic complications: the PVT group, the VTE group and the no thrombosis group. We then assessed for each subgroup the prevalence of liver cirrhosis complications (i.e. all the complications related to portal hypertension, such as: esophageal varices, hepatic encephalopathy, ascites, spontaneous bacterial peritonitis, hepatorenal syndrome) and comorbidities, in particular those included in the most common thrombosis prediction scoring systems for medical inpatients, such as the Padua prediction score (e.g. heart/respiratory failure, acute myocardial infarction/ischemic stroke, acute infection, active cancer) [15]. We intentionally considered HCC separately from the complications related to portal hypertension to better analyze its specific weight on the prevalence of thromboembolic complications. Abdominal surgical operations or invasive procedures performed during the hospital stay were also recorded, the latter including esophageal/gastric varices sclerotherapy or ligation, liver biopsy, non-surgical treatment for HCC, or percutaneous abdominal drainage.

### Statistical analysis

Non-parametric statistics was used due to the non-normal distribution of data, which was confirmed by visual inspection and using the Kolmogorov–Smirnov test. Patients’ characteristics and laboratory examinations were reported as median and interquartile range (continuous variables) or as frequencies and percentages (categorical variables).

Comparisons between the three subgroups of cirrhotic patients according to the presence of PVT, VTE or no thrombosis were performed using the Kruskal–Wallis test and the post hoc analysis with Bonferroni correction for continuous variables, whereas the chi-squared test or the Fisher’s exact test were used for categorical ones, as appropriate (i.e. big contingency tables or when small frequencies were expected, respectively). Finally, the predictive value of age, gender, comorbidities, liver cirrhosis complications, neoplastic disease, abdominal surgery/invasive procedures on the risk of PVT or VTE was evaluated using logistic regression; only variables with a *p* value < 0.1 at univariate analysis were included in the multivariate model.

The analyses were performed with R statistics program version 3.6.2, packages doBy, FSA, nnet. A two-tailed *p* value < 0.05 was considered statistically significant.

## Results

**Fig. 1:**
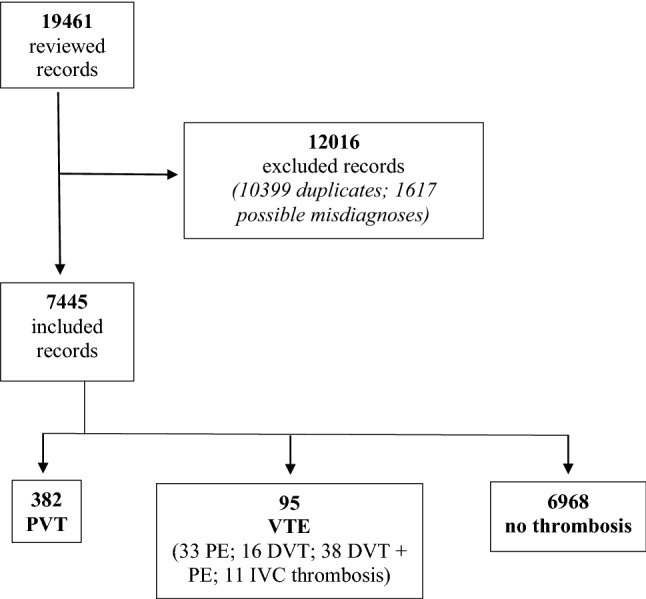
Study flowchart. *PVT* portal vein thrombosis, *VTE* venous thromboembolism, *PE* pulmonary embolism, *IVC* inferior vena cava

An overall number of 19461 electronic discharge records was reviewed. After having removed duplicates and verified the eligibility criteria, 7445 records of patients with liver cirrhosis were kept and further analyzed (Fig. 1).

Patients’ characteristics are described in Table 1. The median age was 64 (54–72) years, with a prevalence of male gender (69.08%). PVT diagnosis was identified in 382 (5.13%) patients, VTE in 95 patients (1.27%). Among patients with VTE, 16 (0.21%) presented DVT, 33 (0.44%) PE, 35 (0.47%) DVT and PE, 11 (0.15%) thrombosis of the IVC. Cirrhotic patients with VTE (67 years [59.5–76.00]) were older than those with PVT (64 years [55.20–71.70] *p* = 0.03) or without thrombosis (63 years [54.00–72.00] *p* = 0.02).

**Table 1:** Demographic and clinical characteristics of the population of cirrhotic patients included in the analysis, stratified according to the presence of portal vein thrombosis (PVT), venous thromboembolism (VTE) or no thrombosis (NT)

	Overall population (*N* = 7445)	PVT group (*N* = 382)	VTE group (*N* = 95)	NT group (*N* = 6968)	*p* value PVT vs NT	*p* value VTE vs NT	*p* value PVT vs VTE
Gender							
Male	5143 (69.08)	276 (72.25)	59 (62.11)	4808 (69.00)	0.19	0.15	0.06
Female	2302 (30.92)	106 (27.75)	36 (37.89)	2160 (31.00)			
Age (years)	64 (54.00–72.00)	64 (55.20–71.70)	67 (59.50–76.00)	63 (54.00–72.00)	0.44	**0.006**	**0.034**
Any liver cirrhosis complication^a^	3006 (40.37)	172 (45.02)	31 (32.63)	2803 (40.22)	0.07	0.14	**0.03**
Ascites	1008 (13.53)	71 (18.58)	13 (13.68)	924 (13.26)	**0.004**	0.87	0.29
Endoscopic signs of portal hypertension	1246 (16.73)	102 (26.70)	12 (12.63)	1132 (16.24)	**< 0.0001**	0.40	**0.003**
HE	591 (7.93)	159 (41.62)	14 (14.73)	418 (5.99)	**< 0.0001**	0.002	**< 0.0001**
SBP	19 (0.25)	–	0 (0.00)	19 (0.27)	0.62	1	1
HRS	84 (1.12)	–	1 (1.05)	83 (1.19)	0.02	1	0.19
Variceal bleeding	396 (5.31)	8 (2.09)	–	388 (5.56)	**0.0015**	**0.0100**	0.37
Any tumor	2036 (27.34)	178 (46.59)	40 (42.10)	1818 (26.09)	**< 0.0001**	**0.0008**	0.49
* HCC*	1524 (20.47)	162 (42.40)	26 (27.36)	1336 (19.17)	**0.0001**	**0.049**	**0.007**
* Other tumors*	596 (8.00)	18 (4.71)	16 (16.84)	562 (8.06)	**0.01**	**0.007**	**0.0001**
Diabetes	1047 (14.06)	80 (20.94)	15 (15.78)	952 (13.66)	**0.0001**	0.55	0.31
Cardiac or respiratory insufficiency	384 (5.15)	14 (3.66)	12 (12.63)	358 (5.13)	0.23	**0.004**	**0.002**
AMI or stroke	22 (0.29)	1 (0.26)	2 (2.10)	19 (0.27)	1	**0.03**	0.10
Acute infection	655 (8.79)	30 (7.85)	21 (22.10)	603 (8.65)	0.64	**< 0.0001**	**0.0002**
Potentially on anticoagulant treatment^b^	166 (2.22)	3 (0.78)	0 (0.00)	163 (2.33)	**0.049**	0.17	1
Abdominal surgery/invasive procedures	1079 (14.49)	101 (26.43)	19 (20.00)	961 (13.79)	**< 0.0001**	0.09	0.23

Continuous variables reported as median and interquartile range, categorical ones as frequencies and percentages. Statistically significant comparisons are highlighted in bold.

*HE* hepatic encephalopathy, *SBP* spontaneous bacterial peritonitis, *HRS* hepatorenal syndrome, *HCC* hepatocellular carcinoma, *AMI* acute myocardial infarction

^a^Excluding HCC and PVT

^b^Atrial flutter/fibrillation, valvulopathies, previous VTE events

### Prevalence of liver cirrhosis complications in patients with PVT or VTE

Any liver cirrhosis complication related to portal hypertension was observed in 45.02% of patients with PVT, in 32.63% of those with VTE (*p* = 0.03) and in 40.22% of those without thrombosis (Table 1). HE (41.6%), endoscopic signs of portal hypertension (26.70%) or ascites (18.58%) were the most commonly observed complications among patients with PVT, with a higher prevalence compared to patients with VTE (14.73% *p* < 0.0001 for HE; 12.63% *p* = 0.003 for endoscopic signs of portal hypertension) or with no thrombosis (5.99% *p* < 0.0001 for HE; 16.24% *p* < 0.0001 for portal hypertension; 13.26% *p* = 0.004 for ascites). Only the prevalence of HE was higher in patients with VTE compared to those without thrombosis (*p* = 0.002). Acute variceal bleeding was more frequent in the group without thrombosis than in PVT patients (5.56% vs 2.09%, *p* = 0.0015), and no case of bleeding was reported in the VTE group (*p* = 0.01). A minority of cases of SBP and HRS were also identified, almost all occurred in the group without thrombosis.

### Prevalence of neoplastic disease in hospitalized cirrhotic patients with PVT or VTE

Any kind of tumor was present in 2036 (27.34%) patients; 1524 (20.47%) were affected by HCC. The prevalence of tumor diagnosis was 46.59% in patients with PVT and 42.10% in those with VTE, respectively, which was a significantly higher percentage compared to patients without thrombosis (26.09%; *p* < 0.0001 and *p* = 0.0008, respectively). HCC was diagnosed in 42.40% of patients with PVT, 27.36% of those with VTE and 19.17% of controls without thrombosis, thus being more frequent in patients with thrombotic events (PVT vs no thrombosis *p* = 0.0001; VTE vs no thrombosis *p* = 0.049) and, among them, mainly in the PVT group than in the VTE group (*p* = 0.007). As expected, neoplastic disease other than HCC was more frequently associated with VTE (16.84%) than with PVT (4.71%; *p* = 0.0001).

### Association between PVT or VTE and comorbidities, surgical operations or invasive procedures

As regards comorbidities, diabetes was diagnosed in 14.06% of the study population, and appeared to be more frequent in patients with PVT (20.94%) than in those without thrombosis (13.66%, *p* = 0.0001). Cardiac or respiratory insufficiency was present in a minority of patients (5.15%) and was more common among patients with VTE (12.63%) than in those with PVT (3.66% *p* = 0.002) or in the no thrombosis group (5.13% *p* = 0.004); similar results were observed for the few cases of AMI or stroke (VTE 2.10% vs no thrombosis 0.27%; *p* = 0.03).

Infection episodes were recorded in 8.79% of the overall cohort; the prevalence was higher in patients with VTE (22.10%) than in those with PVT (7.85% *p* = 0.0002) or in those without thrombosis (8.65% *p* < 0.0001) group.

More than 1000 (14.49%) cirrhotic patients included in this cohort underwent abdominal surgical operations or any invasive procedure during hospitalization, 26.43% in the PVT group, 20.00% in the VTE group and 13.79% in the no thrombosis group, respectively. The frequency was higher in the PVT group than in the no thrombosis group (*p* < 0.0001).

### Risk factors for PVT or VTE in hospitalized cirrhotic patients and impact on outcome

We finally evaluated the impact of demographic factors and comorbid conditions on the risk of thrombotic complications. The univariate analysis showed that HE, HCC or other tumors, abdominal surgery/invasive procedures were significant for both PVT and VTE (Table 2). Ascites, endoscopic signs of portal hypertension, and diabetes increased the risk of PVT, while older age, infections, and cardiovascular complications increased the risk of VTE.

**Table 2:** Univariate and multivariate logistic regression analyses for the prediction of venous thromboembolism (VTE) and portal vein thrombosis (PVT)

	PVT				VTE			
	Univariate		Multivariate		Univariate		Multivariate	
	OR (95% CI)	*p* value	OR (95% CI)	*p* value	OR (95% CI)	*p* value	OR (95% CI)	*p* value
Age	1 (0.99–1.02)	0.17	–	–	1.02 (1–1.04)	**0.006**	1.01 (0.99–1.03)	0.06
LC complications								
Ascites	1.49 (1.14–1.95)	**0.002**	1.25 (0.93–1.69)	0.07	1.04 (0.57–1.87)	0.45	–	–
Endoscopic signs of portal hypertension	1.87 (1.48–2.37)	**< 0.0001**	1.33 (1.02–1.75)	**0.02**	0.74 (0.40–1.37)	0.171	–	–
HE	11.17 (8.91–14.00)	**< 0.0001**	13.98 (10.82–18.06)	**< 0.0001**	2.71 (1.52–4.82)	**0.0003**	3.21 (1.78–5.79)	**< 0.0001**
Tumors								
HCC	3.10 (1.00–2.50)	**< 0.0001**	4.59 (3.60–5.84)	**< 0.0001**	1.58 (1.00–2.5)	**0.02**	1.98 (1.23–3.19)	**0.002**
Other tumors	0.56 (0.35–0.91)	**0.009**	0.76 (0.45–1.26)	0.14	2.3 (1.34–3.98)	**0.001**	2.48 (1.42–4.32)	**0.0007**
Infection	0.89 (0.61–1.31)	0.29	–	–	3.00 (1.83–4.91)	**< 0.0001**	3.01 (1.84–5.05)	**0.0001**
Diabetes	1.67 (1.29–2.16)	**< 0.0001**	1.68 (1.27–2.22)	**0.0001**	1.18 (0.68–2.07)	0.27	–	–
Cardiac or respiratory insufficiency	0.70 (0.40–1.21)	0.10	–	–	2.66 (1.44–4.92)	**0.0009**	2.40 (1.27–4.53)	**0.003**
AMI or stroke	0.96 (1.81–34.27)	0.48	–	–	7.87 (1.81–34.27)	**0.003**	7.86 (1.76–35.12)	**0.003**
Abdominal surgery/invasive procedures	2.24 (1.77–2.85)	**< 0.0001**	2.03 (1.56–2.64)	**< 0.0001**	1.56 (0.94–2.59)	**0.04**	1.24 (0.74–2.08)	0.20

*CI* confidence interval, *OR* odds ratio. Statistically significant comparisons are highlighted in bold.

After variable selection, only endoscopic signs of portal hypertension (OR 1.33 [1.02–1.75] *p* = 0.02), HE (OR 13.98 [10.82–18.06] *p* < 0.0001), HCC (OR 4.59 [3.60–5.84] *p* < 0.0001), diabetes (OR 1.68 [1.27–2.22] *p* = 0.0001) and abdominal surgery/invasive procedures (OR 2.03 [1.56–2.64] *p* < 0.0001) emerged as independent predictors of PVT in the multivariate model (Table 2). HE (OR 3.21 [1.78–5.79] *p* < 0.0001), HCC (OR 1.98 [1.23–3.19] *p* = 0.002) or other tumors (OR 2.48 [1.42–4.32] *p* = 0.0007), and acute illnesses, in particular infections (OR 3.01 [1.84–5.05] *p* = 0.0001), cardiac or respiratory insufficiency (OR 2.40 [1.27–4.53] *p* = 0.003), AMI or stroke (OR 7.86 [1.76–35.12] *p* = 0.003), were the most significant multivariate risk factors for VTE.

Finally, we tested whether PVT or VTE could be associated with length of hospital stay or in-hospital mortality. Although the length of hospitalization was similar among the patient groups (PVT 13 [6–27] days, VTE 14 [6–26] days, no thrombosis 14 [7–25] days; *p* = 0.49), we observed a higher prevalence of death in the group of patients with VTE than in those with PVT or without thrombosis (2.24% vs 0.26% vs 0.50%, respectively; *p* = 0.06). Among all variables tested (complications related to portal hypertension, HCC or other tumors, abdominal surgery/invasive procedures, diabetes, infections, acute illnesses, age) VTE resulted the only independent predictor of mortality (OR 4.45 [1.05–18.81] *p* = 0.042).

## Discussion

The occurrence of PVT and extra-splanchnic thromboembolic complications is insidious and a risk stratification for these severe complications is difficult in patients with liver cirrhosis.

To our knowledge, this is one of the larger retrospective studies in cirrhotic patients, and the only one assessing the prevalence and risk factors for both PVT and VTE in this setting. We found a prevalence of PVT of 5.13% and of VTE of 1.27%, similar to what has already been reported in the literature.

We observed a higher risk of PVT in cirrhotic patients with HE and endoscopic signs of portal hypertension. Coagulation imbalance and local haemodynamic alterations secondary to a “steal effect” through portosystemic collaterals could explain this association. Indeed, slow portal flow velocity, low platelet count, a past history of variceal bleeding or severe portal hypertension, have been recognized as risk factors for PVT [2, 16, 17]. On the contrary, acute variceal bleeding was more frequent in patients without thrombosis, confirming its controversial association with chronic PVT. Overall, these findings suggest that PVT arises in the setting of advanced liver impairment, in contrast to VTE, as previously reported [9, 16, 18, 19].

Interestingly, we found that HE was also an independent predictor of VTE. This could be explained by the effect of a concomitant acute comorbidity, acting as precipitating factor, or by determining prolonged bed rest.

We also confirmed previous evidence that neoplastic disease increases the risk of venous thrombotic complications in cirrhotic patients.

HCC was clearly associated with the risk of PVT and VTE in our study; its thrombotic potential seems to be multifactorial, as HCC can change the haemostatic balance towards a prothrombotic status, and locoregional treatments may predispose to PVT development [20–23]. We observed a HCC prevalence of 42.40% among cirrhotic patients with PVT, and of 27.36% among those with VTE. Overall, we found a PVT prevalence of 10.63% and a VTE prevalence of 1.70% among more than 1500 cirrhotic patients with HCC included in our cohort. This was in line with previously published data [7]; however, the present one represents the largest series including only cirrhotic patients with HCC, considering that the previous studies did not distinguish between patients with liver cirrhosis or those with chronic hepatitis [19, 24, 25]. Although it may be argued that the association between abdominal surgery/invasive procedures and PVT could be attributed to the concomitant presence of HCC, indeed, it was true only in 31.70% of patients. Therefore, as confirmed by the multivariate analysis, abdominal surgery or invasive procedures are independent predictors of PVT even in cirrhotic patients without HCC.

As regards non-HCC tumors, they seem to be associated with the presence of VTE, but not with PVT. This probably highlights the importance of local hemostatic imbalance in the pathogenesis of PVT.

In our cohort, patients with VTE were older and had more cardiovascular comorbidities and infections than controls, although age was not confirmed as a significant predictor in the multivariate model. Increasing age, comorbidities and acute illnesses are well-recognized a risk factors for VTE in both cirrhotic and non-cirrhotic hospitalized patients[9, 26, 27]; systemic inflammation as well as prolonged immobilization could act as prothrombotic triggers in these conditions. Our data are in agreement with those from a recent retrospective cohort of 623 patients with chronic liver disease admitted to intensive care unit, which reported that HCC and sepsis are associated with the occurrence of VTE > 48 h post admission [28].

Interestingly, diabetes mellitus was associated with increased risk of PVT but not of VTE in our cohort. Diabetes and cirrhosis related to non-alcoholic steatohepatitis (NASH) have been reported as independent predictors of PVT [23, 29]. It has been postulated that persistent injury from chronic inflammation in this setting could lead to endothelial cells activation, lipid-derived oxidative injury, necroapoptosis and, ultimately, prothrombotic derangements [30, 31]. It should also be remarked that despite age and comorbidities are generally associated with VTE development, and have been included in several risk assessment models [15, 32], their role in advanced liver disease has yet to be elucidated. Indeed, routine scoring systems are not applicable to cirrhotic patients, as they have been excluded from studies assessing the overall risk of VTE in the general population due to altered coagulation tests and platelet count, that are erroneously perceived as signs of increased bleeding tendency.

Finally, we found a high risk of in-hospital mortality in patients with VTE. Although this result may be affected by the retrospective nature of the study, the consequences of VTE, particularly pulmonary embolism, may explain this outcome.

Even if this is the largest available study reporting the joint prevalence and the risk factors for PVT and VTE in hospitalized cirrhotic patients, it presents several limitations. The use of electronic records can be subjected to misclassification errors. In support of the validity of our study, it is worth mentioning that ICD-9 codes have demonstrated a high accuracy for the identification of cirrhosis [33], HCC [34] and VTE [35, 36]. To reduce coding errors, we included only VTE cases listed as primary and secondary discharge diagnoses, most likely representing acute events, and excluded cases of superficial venous thrombosis and catheter-related venous thrombosis.

Use of administrative records is also subject to bias related to missing information. In particular, the lack of laboratory data precluded liver disease severity estimation, which, however, was deduced from the presence of signs of liver decompensation, and the evaluation of some other risk factors for VTE (e.g. reduced mobility, prothrombotic inherited conditions, obesity, ongoing hormonal treatment). Furthermore, the retrospective nature of the study did not allow identification of data regarding the therapy administered, and particularly which patients might have been anticoagulated or might have received mechanical thromboprophylaxis during hospitalization. Conversely, patients with liver cirrhosis are usually less subjected to anticoagulant therapy even if bedridden, because they are mistakenly considered to be at greater risk of bleeding [37]; this may be a confounding factor that we could not evaluate in this analysis, and which may have led to an increased prevalence of VTE. Finally, our results derive from a population of hospitalized cirrhotic patients, so there may be other risk factors to consider in the outpatient setting.

In conclusion, despite concerns related to acquired increased risk of bleeding, the prevalence of thrombotic events in the setting of liver cirrhosis is not negligible and deserves targeted interventions. Hospitalized patients with HE, portal hypertension, HCC, and diabetes or who are subjected to abdominal surgery/invasive procedures are at increased risk of developing PVT. Elderly cirrhotic patients, those with HE, HCC or other tumors, or acute illnesses show a significant risk of VTE and should receive attention during hospitalization. Future large prospective studies are needed to confirm these results, and to establish whether the population of hospitalized cirrhotic patients could benefit from appropriate thromboprophylaxis.

## Supplementary Information

Below is the link to the electronic supplementary material.

Supplementary file1 (DOCX 16 kb)
