# Supplementary material for: Risk factors for portal vein thrombosis or venous thromboembolism in a large cohort of hospitalized cirrhotic patients
Source: Intern Emerg Med. 2022 Jan 25;17(5):1327–34. doi: 10.1007/s11739-022-02928-8 (PMC9352602; doi:10.1007/s11739-022-02928-8)
Supplement: Supplementary file 1 — Supplementary file1 (DOCX 16 kb) [file 11739_2022_2928_MOESM1_ESM.docx]

**Supplementary Table 1:** ICD-9 codes used to identify diseases and complications.

| **ICD-9 diagnosis code** | **Pathology/procedure** |
| --- | --- |
| 571.2 | Alcoholic cirrhosis of liver |
| 571.5 | Cirrhosis of liver without mention of alcohol |
| 572.2 | Hepatic encephalopathy |
| 572.3 | Portal hypertension |
| 456.0 | Esophageal varices with bleeding |
| 456.1 | Esophageal varices without mention of bleeding |
| 456.2 | Esophageal varices in diseases classified elsewhere |
| 567.23 | Spontaneous bacterial peritonitis |
| 572.4 | Hepatorenal syndrome |
| 789.5 | Ascites |
| 155.0 | Malignant neoplasm of liver, primary (HCC) |
| 452 | Portal vein thrombosis |
| 453.0 | Budd-chiari syndrome |
| 453.2 | Other venous embolism and thrombosis of inferior vena cava |
| 453.4 | Acute venous embolism and thrombosis of unspecified deep vessels of lower extremity |
| 453.8 | Acute venous embolism and thrombosis of other specified veins |
| 415.1 | Pulmonary embolism and infarction |
| 140-209,239 | Malignant neoplasms |
| 428.0, 428.9 | Heart failure |
| 518.81, 618.82 | Respiratory failure |
| 434.91, 434.11, 430, 431, 432.0-432.9, 434.01 | Stroke |
| 410 | Acute myocardial infarction |
| 001-027, 030-041, 090-098, 100-104, 110-112, 114-118, 320, 322, 324-325, 420-421, 451, 461- 465, 481-482, 485-486, 491.21, 494, 510, 513, 540, 541, 562.01, 562.03, 562.11, 562.13, 556-567, 569.5-569.83, 572, 572.1, 575.0, 590, 597, 599, 601, 614-616, 681-683, 686, 711.0, 790.7, 996.6, 998.5, 999.3 | Bacterial or fungal infection |
| 785.52, 995.92 | Septicemia, severe sepsis, and septic shock |
| 42-59, 65-71 | Abdominal surgery |
| 42.33, 42.91, 44.91 | Ligation of esophageal/gastric varices |
| 50.11, 50.13, 50.14 | Liver biopsy |
| 50.23, 50.24, 50.25, 50.26, 99.25 | Locoregional treatments for HCC |
| 54.91 | Percutaneous abdominal drainage |

HCC: hepatocellular carcinoma
